# Neonatal Stridor

**DOI:** 10.1155/2012/859104

**Published:** 2011-12-25

**Authors:** Matija Daniel, Alan Cheng

**Affiliations:** Department of Paediatric Otorhinolaryngology Head & Neck Surgery, The Children's Hospital at Westmead, Sydney Medical School, The University of Sydney, Hawkesbury Road, Westmead, Sydney, NSW 2145, Australia

## Abstract

Neonatal stridor is an important condition, in many cases implying an impending disaster with a very compromised airway. It is a sign that has to be considered with the rest of the history and examination findings, and appropriate investigations should then be undertaken to confirm the source of the noise. Neonates with stridor should be managed in a multidisciplinary setting, by clinicians familiar with the intricate physiology of these children, and with access to the multitude of medical and surgical investigative and therapeutic options required to provide first-rate care.

## 1. Introduction

Neonatal life involves the readaptation of gas exchange from the intrauterine to extrauterine environment. Stridor in this period reflects a critical airway obstruction which may have been anticipated or wholly unexpected. With the advent of better investigations in uterus, greater translation of established therapeutic practices to the neonatal setting, and technological advances that see more children coming to this world at an earlier gestational age, comes the challenge to be more cognizant of the needs of the neonate and to know when to intervene. In modern neonatal intensive care units (NICUs), infants weighing more than 1000 grams and born after 27 weeks of gestation have an approximately 90% chance of survival, and the majority have normal neurological development [1].

Stridor can be defined as a harsh, grating sound as a result of partial obstruction of the laryngotracheal airway. In Latin, originally in the 17th century, it meant “to creak.” Stridor in a neonate potentially implies an impending disaster with a very compromised airway. If seen with significant suprasternal tug and intercostal recession, stridor indicates an airway that may be less than a millimeter away from complete obstruction. Stridor is, however, a sign that has to be considered with the rest of the history and examination findings, and appropriate investigations should then be undertaken to confirm the source of the noise. Its severity, as well as the severity of accompanying respiratory distress, determines the urgency with which investigations are required to proceed, ranging from the well neonate with mild stridor, no respiratory distress, and good feeding, to the one with severe airway compromise requiring immediate intervention.

## 2. Anatomical and Physiological Considerations

The newborn has a much narrower airway in dimensions compared to the infant, child, or adult. The average diameter of the subglottis is around 4.0 mm, and the impact of any form of swelling to an airway that size has been reiterated frequently by quoting the inverse relation of resistance of flow to the radius in a tubular structure (resistance *α*  1/*r* ^4^) [2].

The neonatal respiratory physiology and its impact on airway size highlight the adaptive requirements of the fetus to handle life outside uterus. Their pulmonary blood flow is relatively restricted in uterus by relative fetal hypoxia, but this is radically changed when the establishment of respiration causes improved oxygen content and improved pulmonary blood flow. This can cause the development of unusual blood vessels that extrinsically impress on the airway, as is seen in patients with vascular rings.

The chest wall of the neonate stabilizes a very compliant ribcage, whilst the neonatal lung, especially in a premature child, accounts only for 10–15% of the total lung capacity. To increase the functional residual capacity of the neonatal lung, the child uses (1) expiratory braking—the use of active glottic narrowing during expiration, (2) ongoing active use of inspiratory muscles during expiration, and (3) rapid respiratory rates [3]. Lack of these reflexes, especially in a child with bilateral vocal cord impairment, produces biphasic stridor and rapid decompensation, hence the need for immediate intubation or possible continuous positive airway pressure (CPAP) to maintain a patent airway. However, in a neurologically functioning airway, this physiology also allows tubeless anaesthesia with spontaneous respiration, a common practice when most units perform microlaryngoscopy and bronchoscopy.

## 3. Evaluation of the Neonate

Stridor is but a symptom of an illness that requires a full history and examination. History should cover antenatal and perinatal events, breathing difficulties, feeding, and growth, and previous intubation or intensive care. This has to be complemented by looking for tachypnoea, grunting, inward retractions of the chest wall, nasal flaring, and central cyanosis.

Flexible laryngoscopy is now widely used in the assessment of neonatal stridor. The procedure is well tolerated and can be carried out either via the nose or via the mouth. It gives good view of the supraglottis and vocal cords, allowing one to make an assessment of the dynamic airway in an awake child. However, flexible laryngoscopy does not allow palpation, nor visualization of the subglottis and trachea. Therefore, most children with stridor other than simple mild laryngomalacia still require rigid laryngotracheobronchoscopy.

An additional investigation useful when assessing neonates with airway obstruction is polysomnography. Many babies with airway obstruction will suffer with sleep apnoea as the upper airway musculature relaxes during sleep. Polysomnography can therefore be a useful tool when investigating these neonates and deciding on operative management. It allows the calculation of certain variables such as the respiratory distress index (RDI), frequency and severity of oxygen desaturations (pO_2_), and the retention of carbon dioxide (pCO_2_). If a neonate has values such as RDI >20/hr, frequent pO_2_ desaturations below 90%, or pCO_2_ levels above 50 mmHg, this child may have impending respiratory failure.

The aetiology of stridor in neonates is usually congenital. In a study examining stridor in 219 patients, Holinger confirmed this, also noting that more than half of those children aged under 2.5 years had laryngeal abnormalities [4]. He also found that 45.2% of children with stridor had another associated abnormality involving the respiratory tract, prompting the statement that in evaluating stridor, one does not conclude with laryngoscopy but proceeds with an endoscopic examination of the entire tracheobronchial tree [4].

In a significant proportion of neonatal illnesses, stridor comes as a result of an underlying congenital abnormality probably aggravated by an inflammatory component. It is also important to evaluate the response of stridor to more conservative treatment options. These include the response to adrenaline/epinephrine in croup or subglottic stenosis, the use of positive end expiratory pressure (PEEP) or continuous positive airway pressure (CPAP) for tracheomalacia, the use of inhaled and systemic steroids in inflammation, or the change in body positioning as in mandibular retrognathia.

Narrowing of the laryngotracheal pathway may be at the level of the supraglottis, glottis, subglottis, cervical trachea, or thoracic trachea. It may be a condition extrinsic to these areas, intrinsic to the structures that carry airflow to and from the lungs, or caused by a result of material within the lumen itself. Stridor implies an obstruction at the laryngotracheal airway and has to be distinguished from other airway noises; stertor is a pharyngeal-induced noise which is often worse when the child is asleep (typified by snoring), whilst wheezing is the result of bronchial narrowing. Stridor tends to be worse when the child is awake, feeding, or upset.

There have been many ways of describing the noise heard, including the quality of the sound, the site of obstruction, and the pathological diagnosis. However, the classical description of its relationship to breathing continues to hold firm. Stridor may be inspiratory, expiratory, or both. The length of the expiratory component often allows one to surmise where the site of maximal narrowing or where the closing pressure is most critical. If the sound is purely inspiratory, most otolaryngologists will assume the obstruction is likely supraglottic and the differential diagnosis will likely include laryngomalacia, or something causing the supraglottic structures to draw in as the child inspires. Stridor due to obstruction at the level of the vocal cords or subglottis is often biphasic. Obstruction in the trachea will cause predominantly expiratory stridor, with fixed obstructions (as opposed to dynamic ones) having biphasic stridor.

## 4. Supraglottic Airway Obstruction

Laryngomalacia is the commonest cause of neonatal stridor. It is typified by inspiratory stridor, which worsens with feeding, agitation, and supine positioning. Direct visualization of the larynx typically shows collapse of the supraglottis, tight aryepiglottic folds, an omega-shaped epiglottis, retroflexed epiglottis, and supra-arytenoid tissue prolapse. The condition is thought to be the result of neuromuscular alteration in laryngeal tone with subsequent collapse of the supraglottic structures [5]. Many children with laryngomalacia experience reflux [6]; whilst this may be the result of very negative intrathoracic pressure, reflux itself can contribute to oedema and thus airway compromise.

Laryngomalacia has also been associated with other congenital abnormalities such as neurological problems or Down's syndrome, and up to two thirds may have an associated second airway lesion.

For most children, laryngomalacia is mild and self-resolving [7], with the diagnosis confirmed on flexible laryngoscopy. However, in children with severe or atypical features, rigid airway endoscopy is warranted, with surgical supraglottoplasty being used to relieve symptoms with good results in vast majority of cases.

In the neonatal practice, the other common cause of supraglottic obstruction is the mucus retention cyst in the vallecula. Our unit has seen many of these cases, and the condition is well described in the literature [8]. They often displace the epiglottis posteriorly causing stridor and apparent life-threatening episodes, which is readily corrected with a marsupialisation procedure of the cystic lesion.

## 5. Glottic Airway Obstruction

Glottic obstruction caused by vocal cord motion impairment (VCMI) is the second commonest cause of stridor in a neonate. Whilst bilateral VCMI tends to present with stridor and airway obstruction, patients with unilateral VCMI may also have stridor but additionally present with a weak cry or feeding difficulties due to aspiration. It is important to exclude correctable neural abnormalities resulting from the brainstem, such as the Arnold Chiari malformation, as correction of the pressure to the cerebellar tonsils often resolves a history of fluctuating stridor and airway obstruction. In practice, many cases of unilateral VCMI are iatrogenic, acquired following life-saving intrathoracic procedures such as in tracheoesophageal fistula repair or cardiothoracic procedures for cardiac abnormalities as a neonate. The damaged recurrent laryngeal nerve, often involving the left vocal cord, allows the cord to sit in a paramedian position, which draws in with inspiration. Occasionally, the arytenoid may rotate medially and the vocal cord may be midline and exertional stridor is heard. A variety of treatments are used in unilateral VCMI, including speech and language therapy, vocal cord medialisation, and laryngeal reinnervation [9].

The management of bilateral VCMI centers on achievement of a safe airway. Traditionally, this was achieved with a tracheostomy that may be required in approximately half of patients. However, more recently a variety of open and endoscopic procedures have been used to try and avoid tracheostomy [9]. Botulinum toxin to the cricothyroid muscle, excision of the cricothyroid muscle, vocal cord lateralisation, and endoscopic posterior cricoid split with costal cartilage graft have been proposed at the neonatal or early infancy period, whilst other modalities including vocal cord arytenoidectomy, cordotomy, or recurrent laryngeal nerve reinnervation are for older children with established tracheotomies needing decannulation. It is likely that in two-thirds of children movement in at least one vocal cord will recover in due course, so any aggressive early surgical interventions need to be balanced against the fact that the airway may improve spontaneously, especially as any surgical airway widening may be achieved at the expense of a good voice in the future.

An important diagnosis in VCMI is the differentiation between palsy/paralysis and fixation of the vocal cords due to posterior glottic stenosis. The latter is an acquired problem that has become more frequent in our practice with the increase in the number of extremely premature children requiring ENT input. These children require neonatal intensive care, often having respiratory distress following meconium aspiration, and may require endotracheal intubation for a prolonged period of time. Unfortunately, the endotracheal tube may cause significant glottic irritation, and this in turn causes granulation of the posterior glottis. Acquired subglottic stenosis is rare as a result of better understanding of the pathophysiology of this condition, but this has been replaced to some extent by posterior glottic stenosis. Children born at 24–26 weeks of gestation, weighing less than 1000 g, are renowned for having chronic lung disease, and a small percentage of these neonates appear to react significantly to the presence of the endotracheal tube. It is only with trial extubation that the diagnosis of laryngeal granulomata is made, and these neonates may go on to develop interarytenoid adhesions and, unfortunately, posterior glottic stenosis.

Neurological dysfunction of the laryngeal airway can also come in the form of inflammation at the glottic aperture. The classical description of gastroesophageal reflux disease (GERD) involving the larynx is said to occur when the larynx is bathed in refluxate, and the sensitivity of the larynx is altered [5]. This can present with abnormal constriction of the larynx to stimuli causing intermittent spasmodic constriction of the neonatal larynx, or with lack of constriction allowing significant aspiration of the stomach contents leading to lower respiratory tract symptoms mimicking bronchiolitis or reactive airways disease. Bronchial aspiration confirming lipid-laden macrophages along with signs of the cobblestone tracheal mucosa may assist the pediatrician to consider prolonged antireflux therapy or in some cases fundoplication if maximal therapy fails to prevent ongoing respiratory issues.

Another uncommon cause of glottic obstruction is the laryngeal cleft [10]. This can be missed on routine fibreoptic nasendoscopy and even during laryngoscopy under anesthesia. The gold standard would be to perform a microlaryngoscopy with binocular vision and to spread the posterior laryngeal structures aside and examine the depth of the interarytenoid tissues. Excessive or exuberant tissues in this area can alert one to the possibility. In addition to aspiration, however, children with laryngeal cleft present with stridor when the tissues are more adducted than normal, and inspiration leads to mild indrawing of the vocal folds and the offending noise. Some have suggested that the incidence of laryngeal clefts is increasing, although this may simply reflect greater awareness amongst the clinicians. Small clefts can be repaired endoscopically, but longer ones with a significant cleft between the larynx/trachea and the esophagus require an external approach.

Congenital glottic webs can present as aphonia or a high-pitched cat-like cry along with stridor. Webs can be associated with a genetic alteration as seen with velocardiofacial syndrome [11]. Rarely the web is thin and confined to the larynx alone, and the web may end up being disrupted during intubation, or it can be divided with a sickle knife. More often the web is thick with subglottic extension that appears like a sail on a lateral radiograph. These cannot be treated with simple division, but may require tracheostomy, open repair, keel placement (or the use of perichondrium to prevent web re-forming), and treatment of associated subglottic stenosis. Surgery was previously deemed possible when the child is older, but the improvement of neonatal anaesthesia and microscopic techniques has allowed the performance of this form of surgery at an earlier age, hence avoiding a tracheotomy [11].

Recurrent respiratory papillomatosis is rarely a cause of neonatal stridor, but it can present with normal breathing at birth and then progressive biphasic stridor and loss of voice either in infancy or as a toddler. It is hoped that the recent introduction of vaccines aimed at human papilloma virus (types 6 and 11) will reduce the incidence of this condition [12]. At present, the condition is most commonly treated with repeat debulking using a microdebrider, cold steel, or the CO_2_ laser.

## 6. Subglottic Airway Obstruction

Subglottic stenosis (SGS) is nowadays seen relatively infrequently. If the stenosis is early and soft, a period of laryngeal rest may be used (2-week undisturbed intubation), whilst any granulation tissue can be removed, and any subglottic cysts forming due to obstruction of the mucous glands can be deroofed or removed (using cold steel techniques to minimize tissue damage). If recurrent granulations are a problem, mitomycin C may be of use. Balloon dilation is also useful, but if the oedema is severe or appears to be progressing, then cricoid split (endoscopic or open) can be used. Once the stenosis is firm and established, tracheostomy may be required, with the surgical options of laryngotracheal reconstruction (LTR) or cricotracheal resection (CTR).

To avoid tracheostomy, early surgery has recently been advocated for SGS; a study of patients aged less than 12 months undergoing single-stage LTR found that tracheostomy was avoided in 9 out of 10 neonates and infants [13]. Interestingly, earlier definitive surgical intervention to avoid tracheostomy has also been proposed in the Robin sequence, where distraction osteogenesis and glossopexy avoided tracheostomy in 6 infants that failed CPAP [14]. Similarly, early postnatal surgery has been advocated in children with masses causing airway obstruction. Whilst recent trends towards early surgery may be a tempting way to avoid tracheostomy, it is more technically difficult, with increased physiological risks including those due to blood loss.

Failed extubation is still a common scenario requiring ENT involvement in the neonatal or pediatric intensive care unit. Generally, extubation should be attempted when the child is relatively well, with a leak around the tube. Whilst it is important to ensure that there is no respiratory reason for failed extubation, from the ENT point one should look for both intubation-related and other ENT causes. Close co-operation between the different specialties is required.

Subglottic hemangioma is another condition that has seen its management evolve in the last three years. The child presents with a biphasic stridor, and in 50% of cases, there may be a cutaneous lesion as well. A plain X-ray of the tracheal column sees the classical asymmetrical air column at the subglottis, and they respond fantastically to propranolol such that the operation of tracheotomy for this condition is a thing of the past. There is still controversy about the treatment duration, whether surgical intervention is still required in some cases, and whether simultaneous treatment with systemic steroids is required. However, there is consensus that following endoscopic confirmation of this diagnosis, the premature or low-birth-weight neonate should be closely monitored for hypoglycemic episodes whilst on the treatment with propranolol [15].

## 7. Obstruction in the Trachea

Tracheomalacia is caused by either weakness of the tracheal wall due to alterations in the ratio of cartilage to muscle, or due to hypotonia of trachealis muscle causing anterior prolapsed [16]. It can be primary or secondary to another lesion (such as tracheoesophageal fistula or vascular malformation). Tracheomalacia accompanying tracheal esophageal fistula in the neonate is usually expertly managed by the general pediatric surgeon in our institution. However, children with other associated midline cleft abnormalities such as VATER syndrome need more intensive scrutiny. There have been many case reports over the years where these children have developed tracheal diverticulum that continue to cause significant obstruction despite expert surgical care [17]. Surgical management of tracheomalacia varies depending on site, as the upper tracheomalacia is more amenable to an tracheopexy involving the sternum, whilst lower tracheomalacia involves attachment to the great vessels or possibly even a slide tracheoplasty with primary anastomosis.

Congenital tracheal stenosis with complete tracheal rings is often related to the presence of a pulmonary sling. This sling is a result of the development of a left pulmonary artery coming off the right pulmonary artery, wrapping itself around the trachea at a fetal stage of development. The failure of the development of trachealis or a C-shaped tracheal ring at birth leads to the condition, and if it involves a significant segment of the trachea, the neonate will go on to develop the washing-machine-type stridor characteristic of these cases. The surgical management of these cases often involves relocation of the left pulmonary artery to the pulmonary trunk away from the area of narrowing, whilst correction of the tracheal narrowing can be performed with either a slide tracheoplasty as championed by Grillo [18], or with a variety of other alternatives such as autograft tracheoplasty, pericardial patch tracheoplasty, or stent surgery.

The trachea can also be occluded by vascular abnormalities [19]. A variety of causes have been described. Common vascular rings, completely encircling the trachea, include a double aortic arch and a right-sided aortic arch with aberrant left subclavian artery. Common vascular slings, exerting noncircumferential pressure, are an aberrant innominate artery and a pulmonary artery sling produced by an anomalous left pulmonary artery. Surgical correction of underlying abnormality is often required, but secondary tracheomalacia is also a frequent complication.

## 8. CHAOS and EXIT

Clinical practice has changed much over the last few decades as advances in ultrasonography have facilitated accurate antenatal diagnosis of airway problems and thus appropriate perinatal management. Typical prenatal ultrasound features in congenital high airway obstruction syndrome (CHAOS) include polyhydramnios, dilated trachea and increased echogenicity of the lungs, flat or inverted diaphragm, and ascites [20]. The actual cause of airway obstruction may also be seen. In utero MRI is a useful imaging modality in addition to the ultrasound.

When airway obstruction at delivery is a concern, the procedure of ex utero intrapartum treatment (EXIT) has been used over the last few years to save lives of these children that would have died previously. In addition to EXIT, fetoscopic surgery has also been advocated [21]: creating a perforation in the obstructed larynx allows release of fluid from the obstructed lungs to aid lung development, and although EXIT is still required it is hoped that the long-term respiratory function will be better.

## 9. Conclusion

Stridor in the neonate implies a very severe airway obstruction which needs emergency management. The approach to management needs to be done by clinicians familiar with the intricate physiology of these children who may be still very immature in their development. There are medical and surgical options in the investigative armamentarium, and the use of these newer technological advances may be necessary to temporalize their condition until they grow out of their condition. Importantly, this is a multidisciplinary condition, and good communication among the professionals and carers is likely to achieve long-term successful outcomes for these very vulnerable members of our society.
